# Inhibition of v-*rel*-Induced Oncogenesis through microRNA Targeting

**DOI:** 10.3390/v10050242

**Published:** 2018-05-05

**Authors:** Yongxiu Yao, Yaoyao Zhang, Na Tang, Miriam Pedrera, Zhiqiang Shen, Venugopal Nair

**Affiliations:** 1 The Pirbright Institute & UK-China Centre of Excellence for Research on Avian Diseases, Pirbright, Ash Road, Guildford, Surrey GU24 0NF, UK; zhangyaoyao3848@126.com (Y.Z.); na.tang@pirbright.ac.uk (N.T.); miriam.pedrera@pirbright.ac.uk (M.P.); 2 Binzhou Animal Science and Veterinary Medicine Academy & UK-China Centre of Excellence for Research on Avian Diseases, Binzhou 256600, Shandong, China; bzshenzq@163.com

**Keywords:** microRNA-targeting, inhibition, v-*rel*-induced transformation, hematopoietic specific miRNA

## Abstract

Several studies have shown that microRNA-targeting is an effective strategy for the selective control of tissue-tropism and pathogenesis of both DNA and RNA viruses. However, the exploitation of microRNA-targeting for the inhibition of transformation by oncogenic viruses has not been studied. The v-*rel* oncoprotein encoded by reticuloendotheliosis virus T strain (Rev-T) is a member of the *rel*/NF-κB family of transcription factors capable of transforming primary chicken spleen and bone marrow cells. Here, by engineering the target sequence of endogenous microRNA miR-142 downstream of the v-*rel* gene in a Replication-Competent ALV (avian leukosis virus) long terminal repeat (LTR) with a splice acceptor (RCAS) vector and using a v-*rel*-induced transformation model of chicken embryonic splenocyte cultures, we show that hematopoietic-specific miR-142 can inhibit the v-*rel*-induced transformation, and that this inhibition effect is due to the silencing of v-*rel* expression. The data supports the idea that microRNA-targeting can be used to inhibit viral oncogene-induced oncogenesis.

Avian retroviruses are a group of major pathogens associated with different types of cancers in poultry. Historical studies led by a number of eminent scientists—including Jan Svoboda, whom we are recognizing in this Special Issue—have provided important insights into viruses and cancer [1,2]. Pathogenic avian retroviruses include the different subgroups of avian leukosis viruses (ALV) and their acutely transforming variants such as MC29 and MH2, both of which encode the oncogene v-*myc* [3].

While protein-coding genes such as oncogenes have played a major role in our understanding of viral pathogenesis over the last several decades, non-coding RNAs such as microRNAs (miRNAs) have also recently emerged as major regulators of complex biological processes and diseases [4]. As many miRNAs are expressed in a distinctive cell type-specific manner [5,6,7], artificial miRNA target sites have been utilized to regulate gene expression. Vectors expressing miRNA target sites which are designed to act as a decoy or sponge for the cognate miRNA, thus interfering with the ability of the miRNA to regulate its natural targets, have been used to study the role of miRNAs in cancer, cardiac function, and hematopoiesis [8,9,10,11]. The miRNA targeting of transgene or viral genome by incorporating tissue-specific miRNA target sequences has provided an effective new strategy to improve the specificity and efficacy of gene and stem cell therapy, to reduce the toxicity of oncolytic viruses, and to attenuate viruses to be used in vaccine development [12,13,14,15,16,17]. Initially tested for picornaviruses [14,18], the cell-specific miRNA-dependent suppression of virus replication has proved to be effective *in vivo* for both DNA and RNA viruses [13,16,19,20,21,22].

The avian retrovirus reticuloendotheliosis virus strain T (Rev-T) encodes the v-*rel* oncoprotein, which is a member of the *Rel*/NF-κB transcription factor family. v-*rel* provides a valuable model for studying NF-κB signaling in lymphoid cell cancers because of its ability to transform chicken hematopoeitic cells (12, 15). Previously, we developed a v-*rel*-mediated *in vitro* transformation model of embryonic splenocytes using a Replication-Competent ALV (avian leukosis virus) long terminal repeat (LTR) with a splice acceptor (RCAS) vector [23] that expresses v-*rel* [24]. This represents a very rapid and efficient method to transform chicken splenocytes into cancer cells and immortalized cell lines.

Despite the fact that miRNA targeting has been widely exploited to attenuate or control virus replication, the inhibition effect of miRNA targeting on virus-induced oncogenesis has not yet been studied. miR-142 was first described as a hematopoietic-specific miRNA in mice [25]. Studies have elucidated multiple important regulatory roles of miR-142 in many biological processes and associated signaling pathways during embryonic development, homeostasis, and disease [26]. miR-142 targeting has been used to prevent retrovirally-encoded transgene expression in antigen-presenting cells while allowing long-term transgene expression in other cells [17], as well as to interrupt the picornavirus lifecycle [20]. In the present study, we sought to determine whether the miRNA targeting approach can be used to inhibit oncogenic virus-induced oncogenesis using Rev-T encoded v-*rel*-induced transformation as a model. We first explored the silencing of v-*rel* expression in DF-1 cells by co-infection of RCAS(B) virus expressing miR-142 and RCAS(A) virus expressing v-*rel* with the engineered miR-142 target sequence. We next investigated the inhibition of v-*rel*-induced transformation by endogenous miR-142 in embryonic splenocytes. To determine if v-*rel*-induced transformation can be inhibited by the miRNA targeting approach, we inserted four tandem target sequences of miR-142-3p downstream of the v-*rel* fused with GFP (green fluorescent protein) in RCAS vector RCAS(A)-v-*rel*-GFP by introducing annealed oligos miR-142T-F and miR-142T-R (Table 1) into NotI and SpeI sites, generating RCAS-A-v-*rel*-GFP-142T (Figure 1a). A negative control miRNA [27] target sequence was cloned in the same way using primers miR-NST-F and miR-NST-R (Table 1), generating RCAS-A-v-*rel*-GFP-NST (Figure 1a).

The vector contains the GFP marker which allows the visualization of infected cells. v-*rel* expression is expected to be downregulated in the presence of miR-142. First, we examined the inhibition effect of v-*rel* expression in DF-1 cells. Due to the lack of miR-142 expression in DF-1 cells, we co-infected RCAS(A)-v-*rel*-GFP-miRT (142T or NST) virus together with an miR-142 expression RCAS virus, RCAS(B)-GFP-miR-142 (Figure 1b). The pre-miR-142 sequence was amplified using primers miR-142-F and miR-142-R and inserted downstream of GFP in RCAS(B)-GFP. The expression of v-*rel* and miR-142 by the corresponding virus was confirmed by Western blotting and miRNA qRT-PCR (Taqman), respectively. As shown in Figure 2, v-*rel* is highly expressed when v-*rel*-142T/v-*rel*-NST is used in infections, whereas v-*rel* expression is greatly decreased when the miR-142-expressing virus is co-infected with v-*rel*-142T but not with v-*rel*-NST (Figure 2b). As a negative control, no v-*rel* is expressed in cells infected with miR-142-expressing virus. The expression of miR-142 is confirmed by miRNA Taqman on RNA extracted from the same samples (Figure 2a). miR-155 is not expressed in DF-1. Consistent with the previous finding that v-*rel* [24] induces miR-155 expression, miR-155 is highly expressed in DF-1 cells when v-*rel* is expressed (Figure 2c). The miR-155 level measured by miRNA Taqman in infected DF-1 does correlate with the v-*rel* level, as miR-155 expression is significantly downregulated when v-*rel* is silenced by miRNA targeting (Figure 2c).

Having demonstrated the inhibition of v-*rel* expression through miRNA targeting in DF-1 cells in the presence of miR-142, we next examined the potential inhibition of v-*rel*-induced transformation in embryonic splenocytes. As the mechanisms of v-*rel*-induced transformation have been extensively studied [28,29,30] and the v-*rel*-mediated *in vitro* transformation model of embryonic splenocytes has been established in our laboratory [24], the transformation properties of the cells are not investigated in this study. As described previously [24], we infected embryonic splenocytes isolated from 19-day old embryos with RCAS(A)-v-*rel*-GFP, RCAS(A)-v-*rel*-GFP-142T, or RCAS(A)-v-*rel*-GFP-NST and harvested cells at day 0 (before infection) and days 2, 6, and 14 post-infection for cell counting, Western blotting, and RNA extraction. The uninfected cells were used as a control. As shown in Figure 3a, the cell number increased initially at day 2 and decreased at day 6 post-infection for both infected and uninfected cells. After six days post-infection, uninfected cells and untransformed cells gradually died off and no cells were left at 14 days post-infection, whereas the transformed cells continued proliferating for both RCAS(A)-v-*rel*-GFP and RCAS(A)-v-*rel*-GFP-NST infection groups. The expression of v-*rel*-GFP protein was assessed by Western blotting, using HY87 antibody [31] for v-*rel* expression. As shown in Figure 3b, only the transformed cells at the last time point showed an expected band, whereas the level of v-*rel* expression at other time points was too low to be detected. α-tubulin was used for the loading control. Due to the undetectable level of v-*rel* expression before transformation, we measured the v-*rel* transcript level by RT-PCR with a random primer for reverse transcription and v-*rel* specific primers v-rel-F and v-rel-R for PCR (Figure 3c). Both GFP (GFP-F and GFP-R) and viral specific primers (HA and envA) for ALV-A virus [32] were used as controls. The primer sequences are listed in Table 1. Although the perfectly complementary miR-142-3p targets were inserted, there is no difference at the transcript level between the transformed and untransformed groups, indicating no direct catalytic cleavage of the viral RNA genome and mRNA. However, the result does reflect a similar level of RCAS virus replication between the different groups. Having been unable to detect the difference in v-*rel* protein and transcript levels, next we measured the miR-155 level as an indication of v-*rel* expression, since v-*rel* is able to induce miR-155 expression as reported previously and in Figure 2b [24,33] (Figure 3d). As expected, miR-155 was upregulated after infection by RCAS(A)-v-*rel*-GFP and RCAS(A)-v-*rel*-GFP-NST, whereas the miR-155 level was very low in uninfected cells and RCAS(A)-v-*rel*-GFP-142T infection when the miR-142 target sequence was present. This indicates that v-*rel*-induced transformation is inhibited by miRNA targeting. Compared to miR-155 expression, which was significantly upregulated in the transformed cell groups, miR-142 expression levels did not change much during the time course of the transformation (Figure 3e).

miRNA targeting has been shown to be an efficient means to restrict viral replication or attenuate viral pathogenesis in specific tissues, based on different miRNA expression profiles of different cell lineages [13,19,20,22]. To our knowledge, this is the first study to demonstrate the effective use of miRNA targeting in inhibiting viral-induced transformation. In this study, we explored the ability of miR-142 to inhibit v-*rel*-induced transformation by the insertion of miR-142 target sequences downstream of the v-*rel* gene in RCAS vector. The inhibition effect of v-*rel* expression was first assessed in DF-1 cells by providing miR-142. Indeed, v-*rel*-induced transformation in embryonic splenocyte was inhibited. The result shows that the indicator system in DF-1 cells is an effective and useful way to assess the inhibition of v-*rel*-induced oncogenesis. Unpublished data on the inhibition of oncogenesis induced by the oncogene Meq of Marek’s disease virus (MDV) confirmed the importance of this strategy, although no *in vitro* model of the transformation is available. Meq is the major oncoprotein in MDV-induced tumorigenesis. The evolution of viruses towards greater virulence demonstrates the need to introduce newer vaccines in order to keep up with the rapidly evolving viruses. miRNA targeting by incorporating the miR-142 target sequence downstream of Meq would be an attractive way to attenuate vv+ MDV as a vaccine candidate. Taken together, the results of the present study demonstrate that miRNA targeting can be used to inhibit oncogene v-*rel*-induced transformation *in vitro*. This strategy could be applied for the inhibition of oncogenesis induced by other oncogenic viruses, such as Marek’s disease virus, to develop molecular defined vaccines.

## Figures and Tables

**Figure 1:**
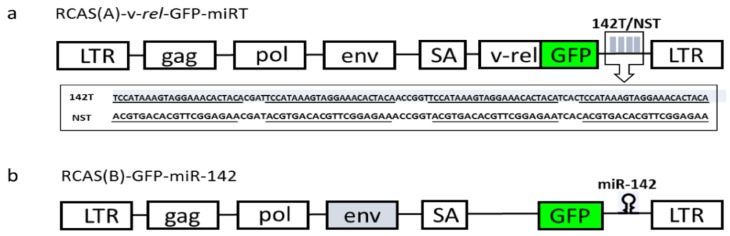
Schematic diagrams of Replication-Competent ALV (avian leukosis virus) LTR with a splice acceptor (RCAS) viruses. LTR, long terminal repeats; GFP, green fluorescent protein; SA, splice acceptor. (**a**) RCAS(A)-v-*rel*-GFP-miRT. Four tandem copies of miR-142-3p or non-silencing miRNA target sequence with the spacer sequences were inserted downstream of GFP; (**b**) RCAS(B)-GFP-miR-142. Pre-miR-142 sequence was inserted downstream of GFP.

**Figure 2:**
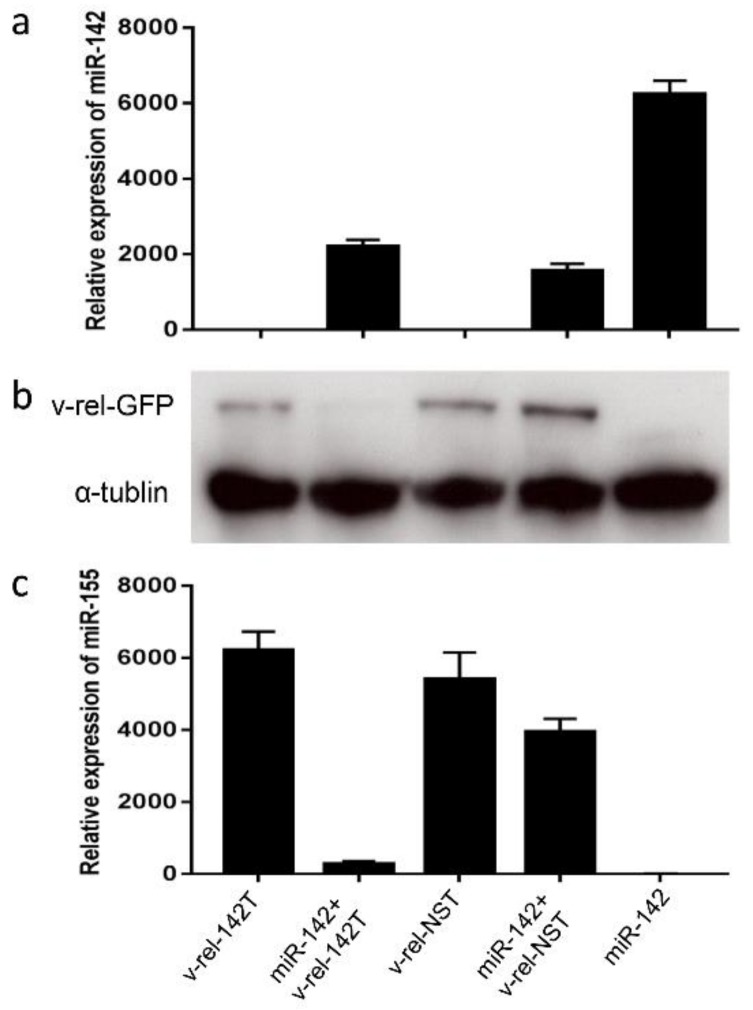
Inhibition of v-*rel* expression through miR-142 targeting in DF-1. Relative expression of miR-142 (**a**) and miR-155 (**c**) measured by qRT-PCR (normalized to let-7a) in RNA extracted from DF-1 infected with RCAS(A)-v-*rel*-GFP-miRT (142T or NST) and RCAS(B)-GFP-miR-142 singly or in combination. Results represent the mean of triplicate assays with error bars showing the standard errors of the mean. (**b**) Cell lysates of the same infected DF-1 cells above were analyzed by Western blot using HY87 antibody for v-*rel* expression. α-tubulin was included as a loading control.

**Figure 3:**
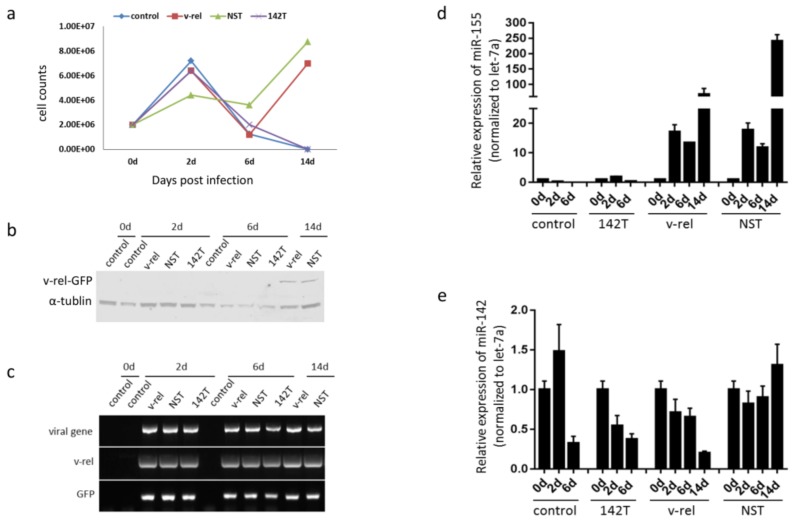
Inhibition of the v-*rel*-induced transformation of embryonic spenocytes through miR-142 targeting. In brief, 2 × 10^6^ 19-day-old embryonic splenocytes were infected with RCAS(A)-v-*rel*-GFP-miRT (142T or NST) or RCAS(A)-v-*rel*-GFP and cells were harvested at days 0, 2, 6, and 14 post-infection for counting, Western blotting, RT-PCR, and miRNA Taqman. (**a**) Cell counts at different time points during the time course of the transformation. (**b**) Cell lysates of infected cells harvested at different time points were analyzed by Western blot using HY87 antibody for v-*rel* expression. α-tubulin was included as a loading control. (**c**) RT-PCR of RNA samples from infected cells using a random primer for reverse transcription and ALV-A-specific primers HA and envA for viral gene detection, v-*rel*-F and v-*rel*-R for v-*rel* detection, and GFP-F and GFP-R for GFP detection. (**d**,**e**) Relative expression of miR-155 and miR-142 measured by qRT-PCR (normalized to let-7a) in RNA extracted from infected cells at different time points. Results represent the mean of triplicate assays with error bars showing the standard errors of the mean.

**Table 1:** List of primers used for the insertion of the miRNA target sequence, miR-142 cloning, and RCAS virus replication detection.

Primer	Sequence (5’–3’)
miR-142T-F	GGCCGCTCCATAAAGTAGGAAACACTACACGATTCCATAAAGTAGGAAACACTACAACCGGTT CCATAAAGTAGGAAACACTACATCACTCCATAAAGTAGGAAACACTACAA
miR-142T-R	GGCCGCACGTGACACGTTCGGAGAACGATACGTGACAC GTTCGGAGAAACCGGTACGTGACACGTTCGGAGAATCA CACGTGACACGTTCGGAGAAA
miR-NST-F	GGCCGCACGTGACACGTTCGGAGAACGATACGTGACAC GTTCGGAGAAACCGGTACGTGACACGTTCGGAGAATCA CACGTGACACGTTCGGAGAAA
miR-NST-R	CTAGTTTCTCCGAACGTGTCACGTGTGATTCTCCGAACG TGTCACGTACCGGTTTCTCCGAACGTGTCACGTATCGTTCTCCGAACGTGTCACGTGC
miR-142-F	GGCCATAATGGCCGGGATGTCCCCTGTGCCCCACTC
miR-142-R	GGCCATAATGGCCAGGCGGCCAGCACAGAACTCCTAC
GFP-F	ATGGTGAGCAAGGGCGA
GFP-R	CCGGTGGTGCAGATGAAC
v-*rel*-F	ATGGACTTTCTCACCAACCTCCG
v-*rel*-R	CGAACGATACCCGACTTG
HA	GGATGAGGTGACTAAGAAAG
envA	AGAGAAAGAGGGGCGTCTAAGGAGA
